# An investigation of the equine epidermal growth factor system during hyperinsulinemic laminitis

**DOI:** 10.1371/journal.pone.0225843

**Published:** 2019-12-05

**Authors:** Melody A. de Laat, Robert J. Spence, Martin N. Sillence, Christopher C. Pollitt

**Affiliations:** 1 Earth, Environmental and Biological Sciences, Queensland University of Technology, Brisbane, Queensland, Australia; 2 School of Veterinary Science, The University of Queensland, Gatton, Queensland, Australia; Massey University, NEW ZEALAND

## Abstract

Equine laminitis is a disease of the digital epidermal lamellae typified by epidermal cell proliferation and structural collapse. Most commonly the disease is caused by hyperinsulinemia, although the pathogenesis is incompletely understood. Insulin can activate the epidermal growth factor (EGF) system in other species and the present study tested the hypothesis that upregulation of EGF receptor (EGFR) signalling is a key factor in laminitis pathophysiology. First, we examined lamellar tissue from healthy Standardbred horses and those with induced hyperinsulinemia and laminitis for EGFR distribution and quantity using immunostaining and gene expression, respectively. Phosphorylation of EGFR was also quantified. Next, plasma EGF concentrations were compared in healthy and insulin-infused horses, and in healthy and insulin-dysregulated ponies before and after feeding. The EGFR were localised to the secondary epidermal lamellae, with stronger staining in parabasal, rather than basal, cells. No change in EGFR gene expression occurred with laminitis, although the receptor showed some phosphorylation. No difference was seen in EGF concentrations in horses, but in insulin-dysregulated ponies mean, post-prandial EGF concentrations were almost three times higher than in healthy ponies (274 ± 90 vs. 97.4 ± 20.9 pg/mL, P = 0.05). Although the EGFR does not appear to play a major pathogenic role in hyperinsulinemic laminitis, the significance of increased EGF in insulin-dysregulated ponies deserves further investigation.

## Introduction

Despite steady progress, and improved clarity around the disease’s causative factors, research into equine laminitis has not yet determined the exact pathophysiological mechanism of this common disease. Laminitis is a painful and costly disease for the equine population worldwide [1]. The outcome of severe laminitis, the distraction of the pedal bone away from the hoof wall, is straightforward to diagnose and understand. However, the determinants of this detachment have proven to be far more difficult to identify [1]. Further, laminitis is a silent disease in the early stages, which reduces the opportunity to investigate the factors that instigate lamellar failure.

Endocrinopathic laminitis is associated with insulin dysregulation, which can occur as persistent or transient, post-prandial hyperinsulinemia, and is the most common form of the disease [2]. The development of a prolonged insulin infusion technique has provided a good experimental model for inducing the disease in otherwise healthy animals [3, 4], enabling detailed investigations of the pathophysiology of endocrinopathic laminitis [5]. Unlike sepsis-related laminitis, a major role for inflammation in disease onset appears unlikely [6, 7]. Instead, hyperinsulinemia produces a more hyperplastic lesion where proliferation and distortion (stretching) of lamellar epidermal basal cells results in lamellar thinning and lengthening [8, 9]. The proliferative component of the response is reminiscent of cancer pathophysiology and suggests a growth factor type role for insulin in the instigation of laminitis. However, studies have found that insulin receptors are not abundant in the lamellae [10], and more importantly that they are not located on the epidermal basal cells [11], thus undermining this hypothesis.

Excess circulating insulin can mediate effects through mechanisms other than binding with the insulin receptor. With respect to laminitis, exploration of the potential effects of insulin on the lamellae have largely converged on a potential interaction between insulin and insulin-like growth factor-1 (IGF-1), or hybrid insulin/IGF-1, receptors [12, 13]. However, recent data are conflicted about this hypothesis [10, 14], prompting us to explore other possibilities. Recently, researchers have demonstrated *in vitro* that insulin can activate the epidermal growth factor receptor (EGFR) [15], and potentiate the effects of epidermal growth factor (EGF) [16, 17]. In the early 1990s, before the identification of insulin as the driving force in endocrinopathic laminitis, a role for the EGFR in chronic laminitis pathophysiology was considered [18]. The presence of the EGFR in the lamellae was confirmed, and they localised receptors to the epidermal basal cells. However, to date no studies have measured EGF concentrations in laminitic horses, or specifically examined EGFRs during insulin-induced laminitis.

Thus, in the present study we tested the hypothesis that insulin potentiates the effects of the EGF system within the lamellae, which may stimulate epidermal basal cell proliferation. Accordingly, we set out to determine whether a synergistic link between insulin and EGF might be instrumental in provoking the onset of endocrinopathic laminitis. The principal aim of this study was to examine the location, quantity and activation state of EGFRs during the developmental and acute stages of insulin-induced laminitis using an experimental model of the disease. A secondary aim of the study was to determine whether there is an association between systemic insulin and EGF concentrations of horses and ponies.

## Materials and methods

### Samples

All samples were collected during two previous experiments that were designed and conducted separately: a study of experimentally-induced hyperinsulinemic laminitis in horses [4] and a study of insulin dysregulation in ponies [19]. The animals were purchased by the university from private owners and operators and were provided with appropriate housing that allowed socialisation and exercise where appropriate, free access to water and a balanced diet that met daily maintenance energy requirements, which was given as two meals (morning and evening). The horses were fed additional hay *ad libitum* during the experimental procedures. Ethical approval for animal use was granted previously by the University of Queensland Production and Companion Animal Ethics Committee (SVS/013/08/RIRDC, QUT/SVS/316/16) and all experiments were performed in accordance with relevant guidelines and regulations of this committee.

#### Tissue samples

Lamellar and skin samples were obtained from the left forelimb of twenty Standardbred horses. Sixteen of the horses received a prolonged euglycemic, hyperinsulinemic clamp (pEHC) in which insulin was administered intravenously (6 μIU/kg/min; Eli Lilly, NSW, Australia) as previously described to induce exogenous hyperinsulinemia [4]. The pEHC was conducted for varying durations to examine the timeline of laminitis development: 6 hours (early developmental time point; n = 4), 12 hours (mid developmental time point; n = 4), 24 hours (late developmental time point; n = 4) or until the animals developed grade 2 lameness [20], which was ~46 hours (n = 4). Each horse treated with a pEHC for ~46 hours was randomly paired with another horse, which was treated with a balanced electrolyte solution for the same period in order to act as a normoinsulinemic control, as previously described [4]. At the conclusion of the infusion period or at the onset of grade 2 lameness (to minimise suffering), each horse was sedated with xylazine hydrochloride (Randlab, NSW, Australia) prior to being euthanased with pentobarbital sodium (Virbac NSW, Australia; both given intravenously), and the lamellar tissue rapidly harvested from the midpoint of the dorsal hoof wall. Duplicate blocks of tissue (5 mm^3^) were processed in two ways: snap frozen in liquid nitrogen (gene expression) and placed in 10% formalin solution for 24 hours (immunohistochemistry). Frozen tissue was transferred to -80°C until use, while the preserved tissue was embedded in paraffin. Tissue samples were not collected during the second study on ponies with naturally-occurring insulin dysregulation.

#### Blood samples

Blood (10 mL) was collected from the control and full-length pEHC Standardbred horses at five time-points for the measurement of two principal EGFR ligands: EGF (receptor agonist) and TGF-β (receptor antagonist). Samples were obtained prior to the infusion, and then again 5, 10, 25 and 45 hours after the infusion commenced. Based on the pEHC (~46 hour animals), and the fact that Standardbred horses are known to be particularly insulin sensitive, all horses were accepted as being metabolically healthy [4]. In order to investigate EGF and TGF-β concentrations during naturally occurring hyperinsulinemia, both ligands were also measured in blood samples (10 mL) collected from ponies before (0 h) and after (2 h) the test meal during an oral glucose test (0.75g/kg bwt dextrose powder in a meal of 200 g bran and 0.3% bwt lucerne chaff). Metabolic status in the ponies was previously determined using an oral glucose test [21] and the cohort included ponies that exhibited exaggerated post-prandial hyperinsulinemia, (n = 9) and ponies without any evidence of insulin dysregulation (n = 7). Including the ponies enabled a comparison between experimental model data (artificially induced hyperinsulinemia) and data from animals with endogenous hyperinsulinemia. The whole blood was placed in a lithium heparin vacutainer that was centrifuged (1500 x *g* x 10 min) to enable immediate harvesting of the plasma. Aliquots (0.5 mL) of all samples had been stored at -80°C since collection.

### EGFR immunohistochemistry

We immunolocalised the EGFR in the digital lamellae of horses without laminitis, and during the developmental and acute stages of laminitis, at the time points outlined above. Human placenta was used as the positive control tissue, and a negative control where no primary antibody was used was also included. Sections of 5μm thickness were cut from the paraffin blocks of tissue and mounted on a charged slide before being deparaffinised with xylene and rehydrated (100, 95 and 70% ethanol for 1 min each, then placed in water). The sections were incubated in antigen retrieval buffer at 125°C for 5 min (pH 6.0) and quenched with 0.3% hydrogen peroxide before blocking with 5% bovine serum albumin. The sections were then incubated overnight at 4°C with the primary antibody (EGFR rabbit anti-human polyclonal sc-373746; Santa Cruz Biotechnology, Dallas, TX, USA; dilution 1:50) in duplicate sections. Reactivity of the antibody with equine tissue was confirmed with western blotting. The sections were washed prior to being incubated with a secondary antibody (mouse anti-rabbit HRP-conjugated; Santa Cruz Biotechnology, Dallas, TX, USA) at room temperature for 2 hours. Staining was developed using DAB substrate-chromogen solution (Sigma-Aldrich, Castle Hill, NSW, Australia) and the slides were counterstained with hematoxylin. The sections were dehydrated before being cover-slipped using DPX mounting medium (Sigma-Aldrich, Castle Hill, NSW, Australia). The slides were randomized prior to blinded examination by a single investigator who examined the full length of the primary epidermal lamellae and reported presence/absence and location of staining.

### EGFR gene expression

For total RNA extraction skin and lamellar tissues (50–100 mg) obtained from control and laminitic (~46 hour time point) horses were homogenized (Omni International, Kennesaw, Georgia, USA) in trizol reagent (1 mL/100 mg tissue) according to the manufacturer’s instructions (Thermo Fisher Scientific, Waltham, Massachusetts, USA). The genomic DNA was eliminated with RNAse-free DNAse I. The RNA concentration and purity were determined with a NanoDrop 2000 (Thermo Fisher Scientific, Waltham, Massachusetts, USA) and integrity assessed with a 2100 Bioanalyzer (Agilent Technologies, Santa Clara, CA), prior to cDNA synthesis using the Tetro cDNA Synthesis Kit (Bioline, Alexandria, NSW, Australia). Each reaction (20 μL) contained 3 ng of RNA, 1 μL Oligo (dT)18, 4 μL of 5x reverse transcription buffer, 1 μL RiboSafe Rnase inhibitor, 1 μL Tetro reverse transcriptase (200 μg/μL) and DEPC-treated water. The cDNA was stored at -80°C until polymerase chain reaction (PCR) analysis. The EGFR primer pair (forward: 5’-TGGACTCATGGACTGGTTTGGC-3’, reverse: 5’-TGGATCAACTCAGTGCAGCAAG-3’) was designed using Primer-BLAST software using available equine sequence data [22]. No template, negative controls containing water instead of cDNA were included. An additional negative control containing pancreas cDNA was included to test for non-specific products. The PCR conditions were optimised using MyTaq HS Red (Bioline, Alexandria, NSW, Australia) in a touchdown PCR protocol described previously [23]. Briefly, reactions (25 μL) comprised 150 ng cDNA, 5 μL 5x MyTaq Reaction Buffer, 0.8 pM/μL each of the forward and reverse primers and 16 μL deionised water (dH2O). Each cycle involved an initial denaturation step at 95°C for 3 min, then 15 cycles of denaturation for 15 sec (95°C) and annealing for 30 sec (65°C decreasing to 50°C) with 30 sec extension (72°C). This was followed by 20 cycles of denaturation for 15 sec (95°C) annealing for 30 sec (50°C) and extension for 30 sec (72°C) and a final extension step for 5 min (72°C). After cycling, the PCR products were visualized (to confirm expected size) with 1.5% agarose gel electrophoresis.

### Droplet digital PCR

To obtain absolute quantification of copy numbers of EGFR in the skin and lamellae of the horses, droplet digital PCR (ddPCR) was performed using EvaGreen Supermix (Bio-Rad, Hercules, CA, USA) on the QX200 Droplet Digital System. Each 22 μL reaction contained 11 μL EvaGreen Supermix 2X, 9.12 μL Ultrapure water (ThermoFisher Scientific, Waltham, Massachusetts, USA), 0.1 μL cDNA and 9.1 pM/μL of each primer (forward and reverse). Droplets were generated according to manufacturer instructions. The touchdown PCR protocol described above was used with a ramp rate of 2°C/sec for ddPCR. Samples were analysed in duplicate.

### Assays

#### Tyrosine kinase assay

The relative phosphorylation of the EGFR in laminitic (pEHC; ~46 hour time point; n = 3 due to insufficient signal in one horse), and non-laminitic (control) horses, was determined using a human, phospho-receptor tyrosine kinase array (ARY001B, R&D Systems, Minneapolis, MN, USA). Each frozen lamellar sample was pulverised with a mallet prior to protein extraction using homogenisation (Omni International, Kennesaw, Georgia, USA) and the cell lysis buffer provided with the assay. The protein concentration of each sample was determined with a bicinchoninic acid (BCA) assay (Thermo Fisher Scientific, Waltham, Massachusetts, USA) and colorimetric detection using a spectrophotometer at 562 nm (GloMax Explorer, Promega, Madison, WI, USA). The assay was carried out according to the manufacturer’s specifications except for an increase in the incubation time of the chemiluminescent reagent to 40 min. The luminescent signal was detected with a ChemiDoc^™^ scanner (BioRad, Hercules, CA, USA). The signal intensity of the phospho-array was determined with the image processing software Fiji [24].

#### ELISAs

Serum insulin concentrations were measured previously by the investigators in serum using either a radioimmunoassay (horse samples; Siemens Healthcare, Victoria, Australia) or at a commercial laboratory (VetPath, WA, Australia) using a chemiluminescent assay (pony samples; Immulite 2000 XPi, Siemens Healthcare, Victoria, Australia) [4, 19]. Both assays were validated in the respective laboratories for use with equine serum. However, as a different assay was used for the horse and pony samples, the insulin concentrations were not compared between groups, but were simply used to correlate insulin and EGF concentrations within each group. The total EGF concentration was measured in plasma with an equine ELISA (MBS015349, Resolving Images, Victoria, Australia), with an intra-assay CV of 9.1%. The plasma TGF-β (major inhibitor of keratinocyte growth) concentration was measured using a human quantikine ELISA (DB100B, R&D Systems, Minneapolis, MN, USA) to try and determine whether there was an imbalance between the stimulation and inhibition of keratinocyte growth during laminitis. This ELISA had been validated for use with equine plasma previously [25], but did not validate adequately in the current study and the data are not reported.

### Statistical analyses

The data were examined for a normal distribution and where normally distributed according to a Shapiro-Wilk test were analysed with parametric tests. Data that did not have a normal distribution were either log transformed to obtain a normal distribution, or analysed with non-parametric tests. All data are reported as mean ± s.e.m or median [interquartile range]. Statistical outliers were determined with Grubb’s test. Significance was set at P < 0.05 with trends reported at P < 0.1. The data analyses were performed with SigmaPlot v.13 (Systat, San Jose, CA, USA) or R v. 3.5 (R Foundation, Vienna, Austria).

Comparisons between horse treatment groups (i.e. control group and pEHC) were made with an unpaired t-test, and between horses and ponies with a Mann-Whitney test. The pre- and post-prandial pony samples were compared with a paired t-test. Investigations of the EGF concentrations in horses over time were undertaken with a repeated-measures ANOVA. Correlations were investigated with Pearson’s co-efficient. Droplets from ddPCR were analysed in an absolute quantitation experiment before the amplitude and cluster data were analysed using the ddpcRquant script [26]. Amplicon counts were converted to mRNA counts per microgram of RNA and analysed using an ANOVA on ranks.

## Results

### EGFR immunohistochemistry

There was abundant positive staining for the EGFR throughout the lamellae both in the control horses, where lamellae had the typical appearance of a healthy dermo-epidermal interface, and of horses in the developmental and acute stages of insulin-induced laminitis. This staining was principally located in the parabasal cells of secondary epidermal lamellae (SEL), but was also present, to a lesser extent, in lamellar basal cells. There was little positive staining of the dermis or dermal structures, such as blood vessels.

#### Control horses

**Fig 1:**
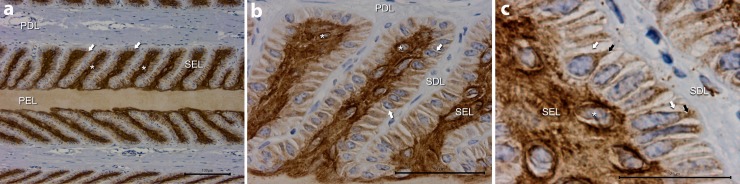
The EFGR immunostaining of lamellae from control (non-laminitic) horses. **A:** The parabasal cells (*) of each secondary epidermal lamella (SEL) showed positive immunostaining. The cytoplasm of lamellar epidermal basal cells (↓), the cell membrane of lamellar basal cells (adjacent to the basement membrane) and the keratinised axis of the primary epidermal lamella (PEL) stained lightly. Staining was absent in the primary dermal lamellae (PDL). **B:** At higher magnification the positive staining of the parabasal cells is diffuse, but particularly evident around nuclei (*). The cytoplasm of lamellar epidermal basal cells stained lightly and was absent adjacent to nuclei (↓) except where it extended to the base of the cell. Staining was absent in secondary dermal lamellae (SDL). **C:** Basal cell staining was absent adjacent to nuclei except where it extended to the cell base (black ↓), contiguous with the dermo-epidermal junction. There was no immunostaining of the basement membrane. A: Bar = 100 μm, B: Bar = 50 μm, C: Bar = 25 μm.

Positive immunostaining was located in the parabasal cells of the SEL (inner core of the SEL, Fig 1A). Immunostaining was also present in the cytoplasm and on the cell membrane of lamellar basal cells (adjacent to the basement membrane). The keratinised axis of primary epidermal lamellae (PEL) stained lightly. Staining was absent in primary dermal lamellae (PDL) and all other dermal tissue.

Higher magnification showed the positive immunostaining of SEL parabasal cells was distributed throughout the cytoplasm and especially concentrated around the nucleus (Fig 1B and 1C). Immunostaining was present in the cytoplasm of lamellar epidermal basal cells except adjacent to the nucleus where its absence formed an incomplete perinuclear halo (Fig 1B and 1C). The basal cell staining extended to the cell base (Fig 1C) and was contiguous with the dermo-epidermal junction. Cell membrane staining was evident consistent with presence of the EGFR on the cell surface of basal cells. There was no staining of the basement membrane.

#### Developmental (6 h, 12 h, 24 hour) and acute (~46 hour) phases

**Fig 2:**
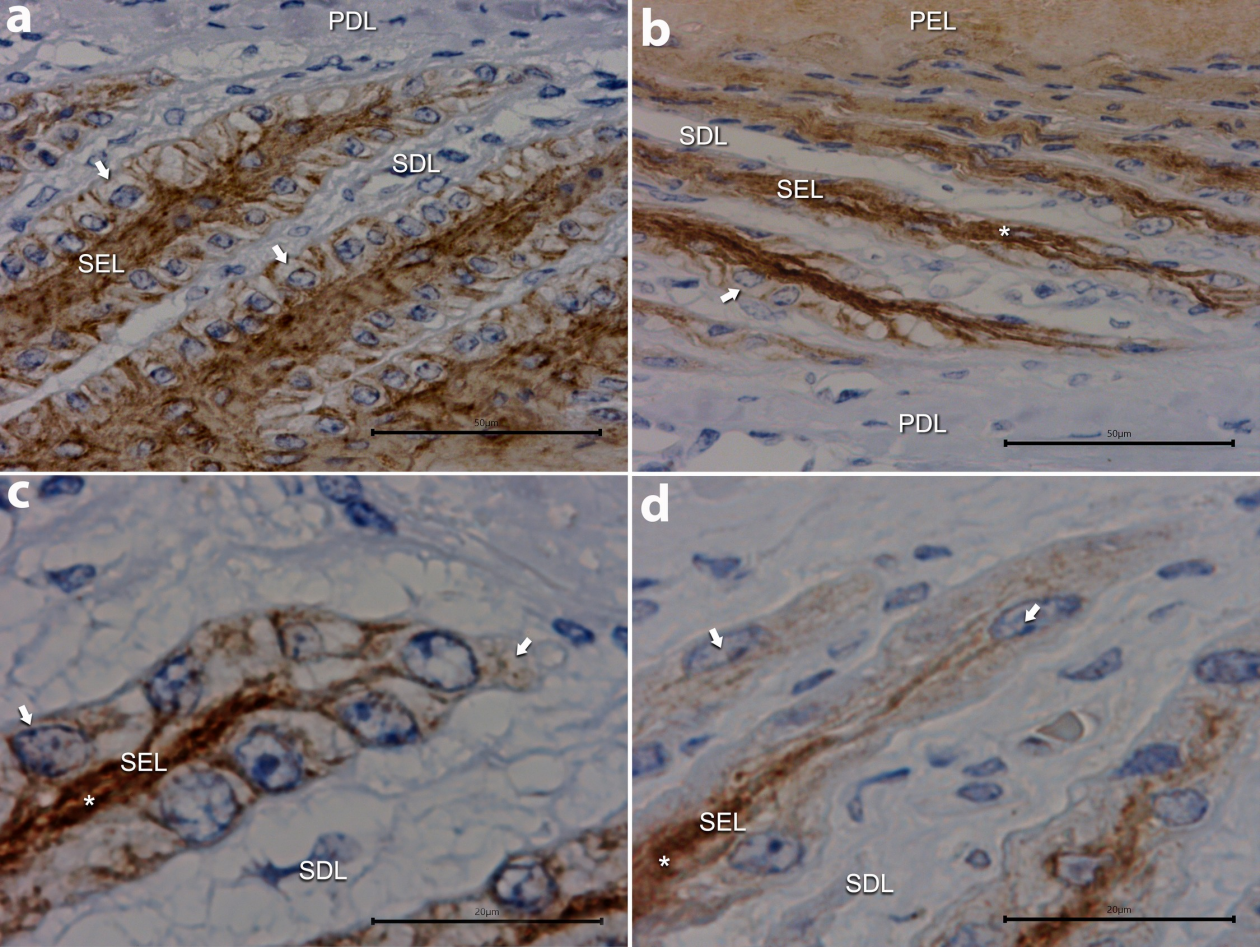
**The EFGR immunostaining of lamellae from horses treated with insulin for 12 (A), 24 (B, C) and ~46 (D) hours. A:** Secondary epidermal lamellae (SEL) are attenuated with pointed instead of rounded tips. The basal cell staining that extended to the cell base in control samples is absent (white ↓). Staining was absent in the primary dermal lamellae (PDL) and secondary dermal lamellae (SDL). **B:** Parabasal cell staining is still present (*). The lamellar attenuation is more marked, with pointed instead of rounded tips. Many basal cell nuclei are oval-shaped but oriented parallel to the SEL axis instead of perpendicular. The cytoplasmic staining that extends to the cell base in control samples is absent (↓). **C:** At higher magnification the rounded basal cell nuclei are situated abnormally close to the basement membrane. The cytoplasmic staining that extends to the cell base in control samples remains absent (↓). **D:** The lamellae are now extremely attenuated and while parabasal cell staining is still present (*), basal cell staining is virtually absent with very mild perinuclear staining (↓) and decreased evidence of cell membrane immunostaining. There was no immunostaining of the basement membrane. A: Bar = 50 μm, B: Bar = 50 μm, C and D: Bar = 20 μm.

Immunostaining at 6 hours was indistinguishable from the control (healthy) samples. For the most part immunostaining at 12 hours was indistinguishable from the control and 6 hour time point samples (Fig 2A), with parabasal cell staining persisting. Structurally, the laminitis lesion had progressed by 24 hours, evident as loss of the normal club shape of the lamellar tips with lamellar attenuation as previously described [8] (Fig 2B). In these lamellae basal cell nuclei were rounded instead of the normal oval shape and were located centrally in the cell. Parabasal cell staining was still present (Fig 2B). The cytoplasmic staining of basal cells that extended to the cell base in control samples was absent (Fig 2B and 2C). During acute laminitis (~46 hour samples; Fig 2D) the lamellae were extremely attenuated with pointed instead of rounded tips as previously reported in this model [4]. Parabasal cell staining was still present (Fig 2D). However, basal cell staining was virtually absent at ~46 hours, which included decreased evidence of cell membrane immunostaining.

### EGFR gene expression and abundance

**Fig 3:**
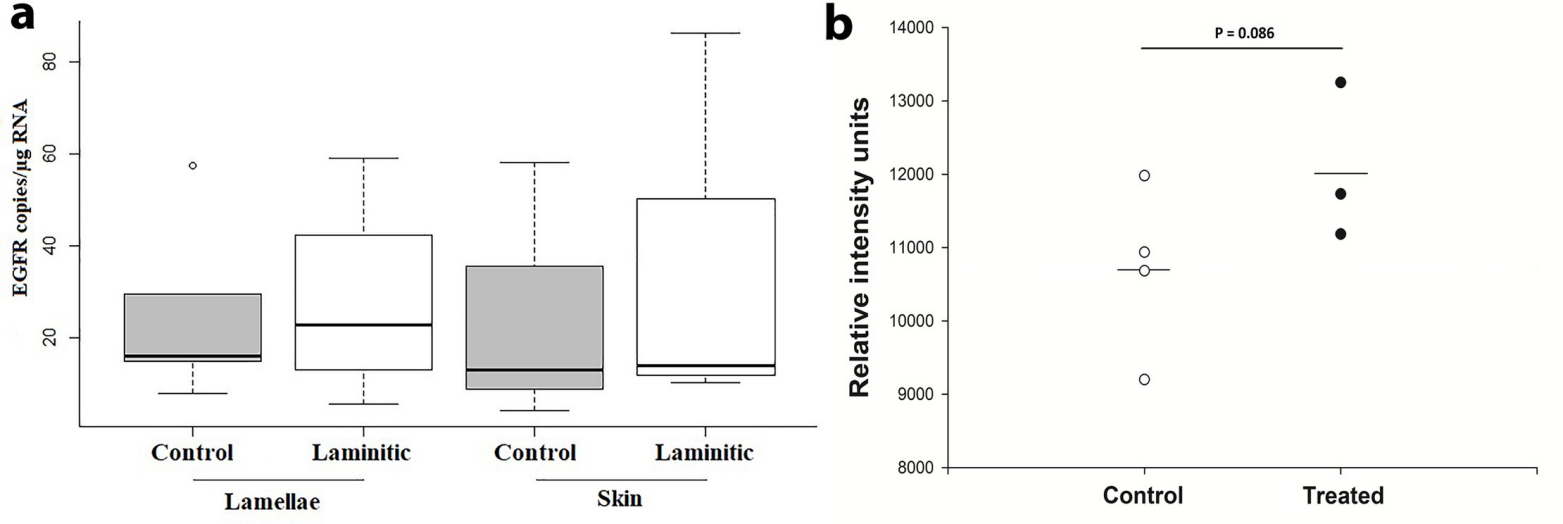
Copy number and phosphorylation of the epidermal growth factor receptor (EGFR). **A:** The median [range] gene copy number per μg of RNA is reported in the lamellae and skin of healthy horses (n = 4), and horses with acute, insulin-induced laminitis (n = 4). Gene copy number was not different between groups or treatments. **B:** The phosphorylation of the EGFR tended to be marginally increased (P = 0.086) in horses with insulin-induced laminitis (●; n = 3), compared to healthy horses (○; n = 4; □ mean).

The EGFR gene was present in both the skin and lamellae of all horses, as expected (S1 Fig). No difference in the abundance of the EGFR gene was found between healthy control horses and horses with acute laminitis (Fig 3A). Further, there was no difference in EGFR abundance in the skin of healthy horses, compared to those with laminitis (Fig 3A).

### EGFR activation

The presence of the phosphorylated state of the EGFR in the lamellae tended to be increased (P = 0.086; one-tailed test; α = 0.39) in horses with acute laminitis, compared to healthy control animals (Fig 3B).

### EGF concentrations

**Fig 4:**
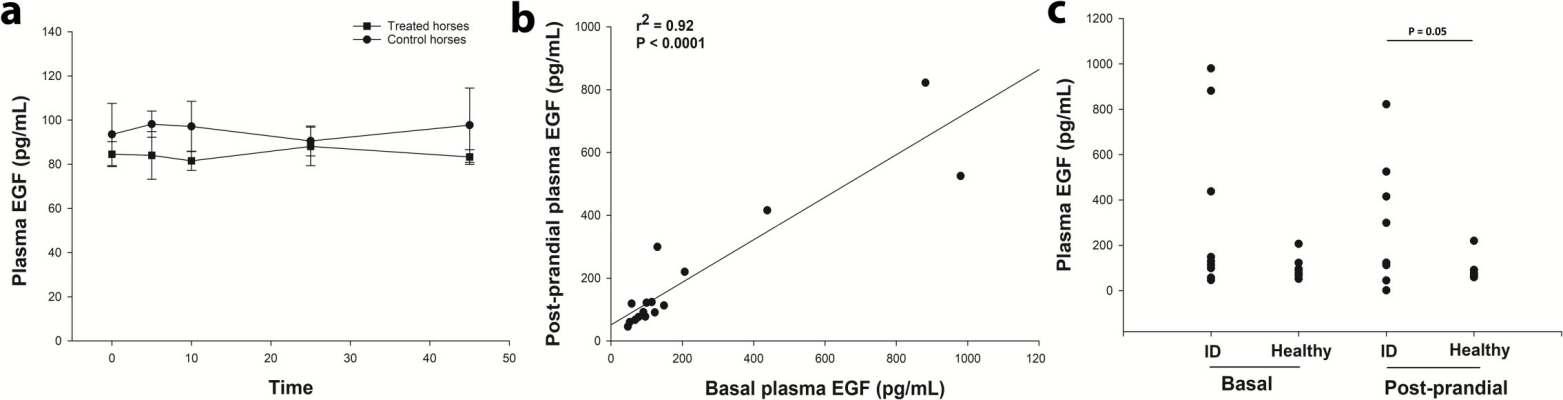
Plasma epidermal growth factor (EGF) concentrations in horses and ponies. **A:** The mean EGF concentrations did not differ between healthy horses (●; n = 4) and horses with insulin-induced laminitis (■; n = 4). **B:** In ponies (n = 16), the basal and post-prandial EGF concentrations were strongly correlated. **C:** When divided into healthy (n = 7) and insulin-dysregulated (ID; n = 9) groups, the ponies with insulin dysregulation tended to have higher basal (P = 0.08) and post-prandial (P = 0.05) EGF concentrations, compared to healthy ponies.

One of the pEHC-treated horses was deemed a statistical outlier (Z = 2.26) with EGF concentrations at least five-fold higher than the rest of the animals, so the data from this animal were ignored. The median, basal EGF concentration of the remaining seven (metabolically healthy) horses was 91.7 [77.3–95.7] pg/mL. The basal EGF concentrations did not correlate with basal insulin concentration in the horses (r^2^ = -0.31; P = 0.50). Further, the EGF concentrations during acute laminitis (~46 hour samples) did not correlate with insulin concentration measured at the same time in these horses (r^2^ = -0.32; P = 0.50), which was not unexpected given the marked exogenous hyperinsulinemia. Lastly, there was no effect of treatment or time on the EGF concentrations in the control and insulin-treated horses (Fig 4A).

The basal plasma EGF concentration was more variable in ponies, than in horses, ranging from 47 to 980 pg/mL. The median concentration was 107 [69.9–192] pg/mL, which did not differ from horses. The post-prandial EGF concentrations in ponies (116 [76.2–280] pg/mL) also did not differ from the pre-prandial concentration. However, the pre- and post-prandial concentrations were correlated (r^2^ = 0.92; P < 0.0001; Fig 4B). While the EGF concentration tended towards being correlated with the serum insulin concentration in basal samples (r^2^ = 0.46; P = 0.076), there was no association in the post-prandial samples (r^2^ = 0.38; P = 0.15).

When the ponies were grouped by insulin regulation status it was found that the median basal EGF concentration did not differ (P = 0.2) between ponies that were insulin-dysregulated (130 [78.5–660] pg/mL), and those that were normally insulin-regulated (90.7 [67.5–123] pg/mL). However, there was a tendency towards a difference (P = 0.08; one-tailed test; α = 0.41) following log transformation of the data. The post-prandial EGF concentrations (these data were normally distributed) in insulin-dysregulated ponies (274 ± 90 pg/mL) were marginally higher (P = 0.05; one-tailed test; α = 0.49), than healthy ponies (97.4 ± 20.9 pg/mL; Fig 4C).

## Discussion

The EGFR is a tyrosine-kinase receptor that is integral in epithelial cell biology. Activation of the receptor mediates cell proliferation, which is why it has been the focus of intense research in the field of cancer therapeutics [27]. Similar to neoplasia, equine laminitis is a disease that involves epidermal cell proliferation, in this case in the epidermal basal cells of the digital lamellae [8, 9]. If the EGFR could be implicated in laminitis pathophysiology an array of new therapeutic options for treating the disease (i.e. EGFR antagonists) could be readily adapted from cancer therapeutics for laminitis treatment. The EGFR and its ligands are overexpressed in many human cancers, and dysregulation of the EGF/R network at multiple levels signals a poor prognosis [28–30]. However, in the current study there was no evidence of an increased expression (neither gene nor protein) of the lamellar EGFR during experimentally-induced laminitis, nor were there increased concentrations of EGF (either during the model or in ponies prone to laminitis), which suggests that the EGF system is not central to the pathogenesis of laminitis. Recently, it was shown that epidermal cell stretching was a key lesion in insulin-induced laminitis in ponies [31], and the current results are supportive of the hypothesis that epidermal cell stretching might be more important than proliferation in laminitis pathology.

The findings from this study confirm those of an earlier study that the EGFR is abundantly expressed in the equine lamellae, with most staining in the SEL [18]. In that study, chronic laminitis was typified by a loss of high affinity binding sites for the EGFR in the lamellae [18]. However, the study [32] did not indicate an overall change in immunolocalistion (using autoradiography) of the EGFR during chronic laminitis, and the current study found that there was no change in total gene expression of the EGFR during acute laminitis, compared to the healthy foot. There are several reasons that might account for these inconclusive outcomes including: 1) different experimental approaches, 2) the comparison of an experimental model with naturally occurring disease, 3) that chronic laminitis has a much longer time course than acute disease and 4) that the type of laminitis in the earlier study was not characterised with respect to the insulin status of the animals. Either way, either a decrease or lack of change in EGFR expression during laminitis does not support the theory that this receptor system is integral to laminitis onset.

The strong EGFR immunoreactivity within the parabasal cells of the SEL was consistent across all sections from all 20 animals examined, but is not consistent with the only other report of EGFR labelling in the lamellae where the basal cells reacted most strongly [32]. In addition, reports of EGFR distribution in equine gastric tissue [33], and epithelial tissues from other species [34] consistently show that the receptor predominates in epidermal basal cells and becomes less apparent in parabasal cells. This distribution pattern is frequently attributed to the higher proliferation rate of basal cells, compared to the more differentiated parabasal layer [35]. The strong staining of the parabasal cells in the current study seems to add further weight to the conclusion that the EGFR is not key to the proliferative lesion of laminitis. As discussed above, an early pathological change in the epidermal cells during insulin-associated laminitis was recently identified to be cell stretching, rather than proliferation [31]. If morphological changes to the basal cells precede the increased rate of mitosis, this might suggest that tissue growth hormones do not instigate the disease, and that another unrelated catalytic factor remains unidentified.

The EGFR might undergo mildly increased activation (phosphorylation) during acute laminitis. Studies *in vitro* have demonstrated that phosphorylation of the EGFR in response to its ligands is transient, and that it is related to the downregulation of the receptor by the ligand [36, 37]. In the current study, a continual cycle of receptor phosphorylation and downregulation could be expected during the persistent hyperinsulinemia induced by the model and is supported by cell surface and cytoplasmic immunostaining, and the mild reduction in basal cell staining during acute disease, as outlined above. It might be that the EGF system has a role in attempted repair of lamellae during laminitis, as EGF is known to be important in wound remodelling [38]. This could help to explain why the degree of phosphorylation was not stronger, although the small sample size (sub-optimal statistical power) is also likely to have affected this result. Overall, these data indicate that although the EGFR may be participating in hyperinsulinemic laminitis pathophysiology, its role is likely to reflect normal physiological responses to the onset of lamellar damage.

Despite extensive searching, we could not find a report of the expected plasma EGF concentration in either horses or ponies, and so we cannot state whether our results fall within the expected range of this hormone in this species. As such, this may be the first report of plasma EGF concentrations in horses and ponies. The basal EGF concentration did not differ between horses and ponies, although all animals were adults and age-related differences may exist (e.g. EGF concentrations decline with age) as they do in other species [39, 40]. The EGF concentration was not correlated with insulin concentration in healthy horses, but this finding was expected due to the extremely low basal insulin concentrations in the horses, which is typical of this breed (lower limit of detection for most animals). However, there was a weak association between basal insulin and EGF in ponies (who generally have greater basal insulin concentrations than horses [41]). This putative association was lost after eating, consistent with a post-prandial surge in insulin, but not EGF, as would be expected. Further examination of an association between EGF and insulin concentrations using a larger cohort of animals would be useful, particularly as a positive relationship might suggest the existence of an insulin-EGF synergy as reported in other species [16].

A comparison of EGF concentrations between ponies that were insulin-dysregulated and those that were not revealed that a relationship between circulating EGF and insulin might occur in ponies when insulin concentrations are high. Because there were only a few animals in this study with marked post-prandial hyperinsulinemia and the comparisons were underpowered, this hypothesis needs to be examined in a larger cohort of animals with insulin dysregulation. Glucose uptake in the small intestine increases with EGF stimulation, through upregulation of the key intestinal sodium-glucose co-transporter (SGLT-1) [42]. We have shown previously that oral glucose bioavailability is increased in ponies with insulin dysregulation, compared to normally regulated ponies [43]. Given the increased circulating EGF in insulin-dysregulated ponies reported here, it is feasible that elevated EGF could be driving increased glucose uptake and insulin-dysregulation in this species. The pathogenesis of equine insulin dysregulation is poorly understood so this theory is worthy of investigation. An increase in EGF during marked hyperinsulinemia could also be associated with the onset of lamellar damage initiated by insulin, with EGF initiating a wound healing response as postulated above. However, a study that specifically examines the relationship between naturally occurring, insulin-associated laminitis and EGF and insulin concentrations is required. As the pEHC is a profound experimental model the exogenous hyperinsulinemia induced during this model in the horses would not necessarily correlate with insulin during laminitis induction, and therefore this study was unable to address this interesting hypothesis.

There are a number of potential ligands for the EGF receptor. In addition to EGF, TGF-α, betacellulin, epiregulin and amphiregulin all bind to the EGFR with varying affinities in other species [44]. These ligands work alone, or together, to achieve activation of the EGFR and catalyse downstream signalling events [36, 45]. The soluble forms of these ligands are all released locally, with metalloproteases from the ‘a disintegrin and metalloprotease’ (ADAM) group important in their processing [46]. Although it has been demonstrated previously that ADAM-TS4 was not upregulated during the onset of insulin-induced laminitis [47], other studies showed that members of the ADAM family were upregulated during laminitis induction [48, 49] so enhanced cleavage of these ligands may be occurring during laminitis onset. There are currently no equine-specific assays for measuring the other EGFR ligands in horses. However, in other species they mediate a host of effects by signalling through the EGFR and can act in an autocrine, paracrine and endocrine manner [35]. Thus, given the diffuse distribution of the EGFR in the lamellae an in-depth analysis of their role in the lamellar epidermis would no doubt be instrumental in improving our understanding of this complex structure.

This study has shown that the EGFR is unlikely to be a pathogenic factor in insulin-associated laminitis pathophysiology, but that it might play a role, at least in part, in epidermal repair. Whether these data are also applicable to other forms of laminitis, such as the inflammatory and septic variants, is unknown. Based on the data reported here, we surmise that laminitis pathophysiology is a multifactorial event, with the involvement of numerous pathways and factors. As a result, we have farther to go with respect to understanding this disease. An important adjunct finding of this study was evidence to support that a potentially synergistic relationship between insulin and EGF exists in horses. Our understanding of equine insulin dysregulation in limited, but the disease is common and is a significant risk factor for laminitis [50]. The finding that EGF is increased in insulin-dysregulated ponies has identified a direction for future research into metabolic dysfunction.

## Supporting information

S1 Fig Agarose gel electrophoresis of epidermal growth factor receptor (EGFR) gene expression in equine lamellae and skin. Lamellar samples (lanes 2–10) and skin (SK, lanes 11–12) of healthy horses (CH1-5) and horses treated with a prolonged euglycemic, hyperinsulinemic clamp to induce hyperinsulinemia and laminitis (TH1-4) were examined for gene expression. A no template control (NTC, lane 13) and tissue not expected to contain the EGFR (-ve, lane 14) was included, as was a base pair ladder (L, lane 1) for identification of an appropriate-sized product.
(PDF)

S1 File Raw data file. (PDF)

## References

[1] Sloet van Oldruitenborgh‐Oosterbaan MM. Laminitis in the horse: A review. Veterinary Quarterly. 1999;21(4):121–7. 10.1080/01652176.1999.9695006 10568001

[2] Karikoski NP, Horn I, McGowan TW, McGowan CM. The prevalence of endocrinopathic laminitis among horses presented for laminitis at a first-opinion/referral equine hospital. Domest Anim Endocrinol. 2011;41(3):111–7. Epub 2011/06/24. 10.1016/j.domaniend.2011.05.004 . 21696910

[3] Asplin KE, Sillence MN, Pollitt CC, McGowan CM. Induction of laminitis by prolonged hyperinsulinaemia in clinically normal ponies. Veterinary Journal. 2007;174:530–5. WOS:000251543700015. 10.1016/j.tvjl.2007.07.003 17719811

[4] de Laat MA, McGowan CM, Sillence MN, Pollitt CC. Equine laminitis: Induced by 48 h hyperinsulinaemia in Standardbred horses. Equine Veterinary Journal. 2010;42(2):129–35. 10.2746/042516409X475779 ISI:000274410200007. 20156248

[5] Patterson-Kane JC, Karikoski NP, McGowan CM. Paradigm shifts in understanding equine laminitis. The Veterinary Journal. 2018;231(Supplement C):33–40. 10.1016/j.tvjl.2017.11.011. 29429485

[6] de Laat MA, van Eps AW, McGowan CM, Sillence MN, Pollitt CC. Equine Laminitis: Comparative Histopathology 48 hours after Experimental Induction with Insulin or Alimentary Oligofructose in Standardbred Horses. Journal of Comparative Pathology. 2011;145(4):399–409. Epub Mar 22 2011. 10.1016/j.jcpa.2011.02.001 21429503

[7] Burns TA, Watts MR, Weber PS, McCutcheon LJ, Geor RJ, Belknap JK. Laminar inflammatory events in lean and obese ponies subjected to high carbohydrate feeding: Implications for pasture-associated laminitis. Equine Vet J. 2015;47(4):489–93. Epub 2014/06/26. 10.1111/evj.12314 . 24963607

[8] de Laat MA, Patterson-Kane JC, Pollitt CC, Sillence MN, McGowan CM. Histological and morphometric lesions in the pre-clinical, developmental phase of insulin-induced laminitis in Standardbred horses. Veterinary Journal. 2013;195(3):305–12. Epub 2012/08/14. 10.1016/j.tvjl.2012.07.003 . 22884985

[9] Karikoski NP, McGowan CM, Singer ER, Asplin KE, Tulamo RM, Patterson-Kane JC. Pathology of Natural Cases of Equine Endocrinopathic Laminitis Associated With Hyperinsulinemia. Veterinary pathology. 2015;52(5):945–56. Epub 2014/09/19. 10.1177/0300985814549212 . 25232034

[10] Nanayakkara SN, Rahnama S, Harris PA, Anderson ST, de Laat MA, Bailey S, et al Characterization of insulin and IGF-1 receptor binding in equine liver and lamellar tissue: implications for endocrinopathic laminitis. Domest Anim Endocrinol. 2019;66:21–6. Epub 2018/09/12. 10.1016/j.domaniend.2018.05.008 . 30205269

[11] Burns TA, Watts MR, Weber PS, McCutcheon LJ, Geor RJ, Belknap JK. Distribution of insulin receptor and insulin-like growth factor-1 receptor in the digital laminae of mixed-breed ponies: An immunohistochemical study. Equine Vet J. 2013;45(3):326–32. Epub 2012/08/29. 10.1111/j.2042-3306.2012.00631.x . 22924550

[12] de Laat MA, Pollitt CC, Kyaw-Tanner MT, McGowan CM, Sillence MN. A potential role for lamellar insulin-like growth factor-1 receptor in the pathogenesis of hyperinsulinaemic laminitis. Veterinary Journal. 2013;197(2):302–6. Epub 2013/02/12. 10.1016/j.tvjl.2012.12.026 . 23394844

[13] Kullmann A, Weber PS, Bishop JB, Roux TM, Norby B, Burns TA, et al Equine insulin receptor and insulin-like growth factor-1 receptor expression in digital lamellar tissue and insulin target tissues. Equine Vet J. 2016;48(5):626–32. Epub 2015/06/23. 10.1111/evj.12474 . 26095356

[14] Baskerville CL, Chockalingham S, Harris PA, Bailey SR. The effect of insulin on equine lamellar basal epithelial cells mediated by the insulin-like growth factor-1 receptor. PeerJ. 2018;6:e5945–e. 10.7717/peerj.5945 . 30519508 PMC6275117

[15] Shin M, Yang EG, Song HK, Jeon H. Insulin activates EGFR by stimulating its interaction with IGF-1R in low-EGFR-expressing TNBC cells. BMB reports. 2015;48(6):342–7. Epub 2014/10/25. 10.5483/BMBRep.2015.48.6.157 25341922 PMC4578621

[16] Chong MP, Barritt GJ, Crouch MF. Insulin potentiates EGFR activation and signaling in fibroblasts. Biochem Biophys Res Commun. 2004;322(2):535–41. Epub 2004/08/25. 10.1016/j.bbrc.2004.07.150 . 15325263

[17] Borisov N, Aksamitiene E, Kiyatkin A, Legewie S, Berkhout J, Maiwald T, et al Systems-level interactions between insulin-EGF networks amplify mitogenic signaling. Molecular systems biology. 2009;5:256 Epub 2009/04/10. 10.1038/msb.2009.19 19357636 PMC2683723

[18] Grosenbaugh DA, Hood DM, Amoss MS Jr., Williams JD. Characterisation and distribution of epidermal growth factor receptors in equine hoof wall laminar tissue: comparison of normal horses and horses affected with chronic laminitis. Equine Vet J. 1991;23(3):201–6. Epub 1991/05/11. 10.1111/j.2042-3306.1991.tb02755.x . 1884702

[19] Fitzgerald DM, Walsh DM, Sillence MN, Pollitt CC, de Laat MA. Insulin and incretin responses to grazing in insulin-dysregulated and healthy ponies. Journal of veterinary internal medicine. 2019;33(1):225–32. 10.1111/jvim.15363 MEDLINE:30506731. 30506731 PMC6335545

[20] Obel N. Studies on the Histopathology of Acute Laminitis. Dissertation: Almqvist and Wiksells Boktryckeri AB, Uppsala, Sweden 1948.

[21] Fitzgerald DM, Walsh DM, Sillence MN, Pollitt CC, Laat MA. Insulin and incretin responses to grazing in insulin-dysregulated and healthy ponies. Journal of Veterinary Internal Medicine. 2019;33:225–32. 10.1111/jvim.15363 30506731 PMC6335545

[22] Ye J, Coulouris G, Zaretskaya I, Cutcutache I, Rozen S, Madden T. Primer-BLAST: A tool to design target-specific primers for polymerase chain reaction. BMC Bioinformatics. 2012;13:134 10.1186/1471-2105-13-134 22708584 PMC3412702

[23] de Laat MA, Fitzgerald DM, Sillence MN, Spence RJ. Glucagon-like peptide-2: A potential role in equine insulin dysregulation. Equine Vet J. 2018;50(6):842–7. Epub 2018/03/05. 10.1111/evj.12825 . 29502360

[24] Schindelin J, Arganda-Carreras I, Frise E, Kaynig V, Longair M, Pietzsch T, et al Fiji: an open-source platform for biological-image analysis. Nature methods. 2012;9(7):676–82. Epub 2012/06/30. 10.1038/nmeth.2019 22743772 PMC3855844

[25] Hauschild G, Geburek F, Gosheger G, Eveslage M, Serrano D, Streitburger A, et al Short term storage stability at room temperature of two different platelet-rich plasma preparations from equine donors and potential impact on growth factor concentrations. BMC veterinary research. 2017;13(1):7 Epub 2017/01/07. 10.1186/s12917-016-0920-4 28056978 PMC5216599

[26] Trypsteen W, Vynck M, De Neve J, Bonczkowski P, Kiselinova M, Malatinkova E, et al ddpcRquant: threshold determination for single channel droplet digital PCR experiments. Analytical and bioanalytical chemistry. 2015;407(19):5827–34. Epub 2015/05/30. 10.1007/s00216-015-8773-4 . 26022094

[27] Holbro T, Hynes NE. ErbB receptors: directing key signaling networks throughout life. Annual review of pharmacology and toxicology. 2004;44:195–217. Epub 2004/01/28. 10.1146/annurev.pharmtox.44.101802.121440 . 14744244

[28] Liu R, Li W, Tao B, Wang X, Yang Z, Zhang Y, et al Tyrosine phosphorylation activates 6-phosphogluconate dehydrogenase and promotes tumor growth and radiation resistance. Nat Commun. 2019;10(1):991 Epub 2019/03/03. 10.1038/s41467-019-08921-8 30824700 PMC6397164

[29] Majumder A, Ray S, Banerji A. Epidermal growth factor receptor-mediated regulation of matrix metalloproteinase-2 and matrix metalloproteinase-9 in MCF-7 breast cancer cells. Mol Cell Biochem. 2019;452(1–2):111–21. Epub 2018/08/04. 10.1007/s11010-018-3417-6 . 30074136

[30] Selemetjev S, Bartolome A, Isic Dencic T, Doric I, Paunovic I, Tatic S, et al Overexpression of epidermal growth factor receptor and its downstream effector, focal adhesion kinase, correlates with papillary thyroid carcinoma progression. International journal of experimental pathology. 2018;99(2):87–94. Epub 2018/04/18. 10.1111/iep.12268 29665129 PMC6031876

[31] Karikoski NP, Patterson-Kane JC, Asplin KE, McGowan TW, McNutt M, Singer ER, et al Morphological and cellular changes in secondary epidermal laminae of horses with insulin-induced laminitis. American Journal of Veterinary Research. 2014;75(2):161–8. WOS:000330926500007. 10.2460/ajvr.75.2.161 24471752

[32] Grosenbaugh DA, Amoss MS, Hood DM, Williams JD. EGF receptor-binding activity in the urine of normal horses and horses affected by chronic laminitis. Domest Anim Endocrinol. 1990;7(3):277–89. Epub 1990/07/01. 10.1016/0739-7240(90)90034-w . 2390863

[33] Jeffrey SC, Murray MJ, Eichorn ES. Distribution of epidermal growth factor receptor (EGFr) in normal and acute peptic-injured equine gastric squamous epithelium. Equine Vet J. 2001;33(6):562–9. Epub 2001/11/27. 10.2746/042516401776563481 . 11720027

[34] Hansen LA, Woodson RL 2nd, Holbus S, Strain K, Lo YC, Yuspa SH. The epidermal growth factor receptor is required to maintain the proliferative population in the basal compartment of epidermal tumors. Cancer research. 2000;60(13):3328–32. Epub 2000/07/26. . 10910032

[35] Schneider MR, Werner S, Paus R, Wolf E. Beyond Wavy Hairs: The Epidermal Growth Factor Receptor and Its Ligands in Skin Biology and Pathology. The American Journal of Pathology. 2008;173(1):14–24. 10.2353/ajpath.2008.070942 18556782 PMC2438281

[36] Macdonald-Obermann JL, Pike LJ. Different epidermal growth factor (EGF) receptor ligands show distinct kinetics and biased or partial agonism for homodimer and heterodimer formation. The Journal of biological chemistry. 2014;289(38):26178–88. Epub 2014/08/03. 10.1074/jbc.M114.586826 25086039 PMC4176247

[37] Gross SM, Rotwein P. Mapping growth-factor-modulated Akt signaling dynamics. Journal of cell science. 2016;129(10):2052–63. Epub 2016/04/06. 10.1242/jcs.183764 27044757 PMC4878993

[38] Cheon SS, Nadesan P, Poon R, Alman BA. Growth factors regulate beta-catenin-mediated TCF-dependent transcriptional activation in fibroblasts during the proliferative phase of wound healing. Experimental cell research. 2004;293(2):267–74. Epub 2004/01/20. 10.1016/j.yexcr.2003.09.029 . 14729464

[39] Valiathan R, Ashman M, Asthana D. Effects of Ageing on the Immune System: Infants to Elderly. Scandinavian journal of immunology. 2016;83(4):255–66. Epub 2016/01/26. 10.1111/sji.12413 . 26808160

[40] Evanson JR, Guyton MK, Oliver DL, Hire JM, Topolski RL, Zumbrun SD, et al Gender and age differences in growth factor concentrations from platelet-rich plasma in adults. Military medicine. 2014;179(7):799–805. Epub 2014/07/09. 10.7205/MILMED-D-13-00336 . 25003868

[41] de Laat MA, Sillence MN, Reiche DB. Phenotypic, hormonal, and clinical characteristics of equine endocrinopathic laminitis. Journal of Veterinary Internal Medicine. 2019;0(0). 10.1111/jvim.15419 30697823 PMC6524085

[42] Hardin JA, Wong JK, Cheeseman CI, Gall DG. Effect of luminal epidermal growth factor on enterocyte glucose and proline transport. The American journal of physiology. 1996;271(3 Pt 1):G509–15. Epub 1996/09/01. 10.1152/ajpgi.1996.271.3.G509 . 8843777

[43] de Laat MA, McGree JM, Sillence MN. Equine hyperinsulinemia: investigation of the enteroinsular axis during insulin dysregulation. American Journal of Physiology—Endocrinology and Metabolism. 2016;310(1):E61–E72. Epub Nov 3, 2015. 10.1152/ajpendo.00362.2015 26530154

[44] Singh B, Carpenter G, Coffey RJ. EGF receptor ligands: recent advances. F1000Research. 2016;5:F1000 Faculty Rev-2270. 10.12688/f1000research.9025.1 . 27635238 PMC5017282

[45] Bergeron JJ, Di Guglielmo GM, Dahan S, Dominguez M, Posner BI. Spatial and Temporal Regulation of Receptor Tyrosine Kinase Activation and Intracellular Signal Transduction. Annual review of biochemistry. 2016;85:573–97. Epub 2016/03/30. 10.1146/annurev-biochem-060815-014659 . 27023845

[46] Zunke F, Rose-John S. The shedding protease ADAM17: Physiology and pathophysiology. Biochimica et biophysica acta Molecular cell research. 2017;1864(11 Pt B):2059–70. Epub 2017/07/15. 10.1016/j.bbamcr.2017.07.001 . 28705384

[47] de Laat MA, Kyaw-Tanner MT, Nourian AR, McGowan CM, Sillence MN, Pollitt CC. The developmental and acute phases of insulin-induced laminitis involve minimal metalloproteinase activity. Veterinary Immunology and Immunopathology. 2011;140(3–4):275–81. 10.1016/j.vetimm.2011.01.013 21333362

[48] Coyne MJ, Cousin H, Loftus JP, Johnson PJ, Belknap JK, Gradil CM, et al Cloning and expression of ADAM-related metalloproteases in equine laminitis. Veterinary Immunology and Immunopathology. 2009;129(3–4):231–41. 10.1016/j.vetimm.2008.11.022 WOS:000266118700015. 19131116 PMC2907504

[49] Wang L, Pawlak E, Johnson PJ, Belknap JK, Alfandari D, Black SJ. Effects of cleavage by a disintegrin and metalloproteinase with thrombospondin motifs-4 on gene expression and protein content of versican and aggrecan in the digital laminae of horses with starch gruel-induced laminitis. American journal of veterinary research. 2012;73(7):1047–56. 10.2460/ajvr.73.7.1047 . 22738057 PMC3535458

[50] Durham AE, Frank N, McGowan CM, Menzies-Gow NJ, Roelfsema E, Vervuert I, et al ECEIM consensus statement on equine metabolic syndrome. Journal of Veterinary Internal Medicine. 2019;33(2):335–49. 10.1111/jvim.15423 30724412 PMC6430910

