# Supplementary figures and images for: An investigation of the equine epidermal growth factor system during hyperinsulinemic laminitis
Source: PLoS One. 2019 Dec 5;14(12):e0225843. doi: 10.1371/journal.pone.0225843 (PMC6894753; doi:10.1371/journal.pone.0225843)

**L CH1 CH2 CH3 CH4 CH5 TH1 TH2 TH3 TH4 C-SK T-SK NTC -VE**

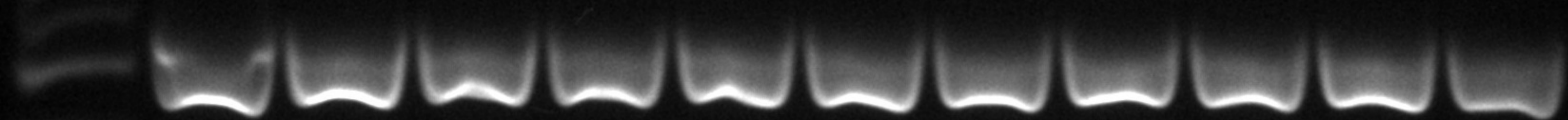

Supplement: S1 Fig — Lamellar samples (lanes 2–10) and skin (SK, lanes 11–12) of healthy horses (CH1-5) and horses treated with a prolonged euglycemic, hyperinsulinemic clamp to induce hyperinsulinemia and laminitis (TH1-4) were examined for gene expression. A no template control (NTC, lane 13) and tissue not expected to contain the EGFR (-ve, lane 14) was included, as was a base pair ladder (L, lane 1) for identification of an appropriate-sized product. (PDF) [file pone.0225843.s001.pdf]
