# Supplementary material for: An investigation of the equine epidermal growth factor system during hyperinsulinemic laminitis
Source: PLoS One. 2019 Dec 5;14(12):e0225843. doi: 10.1371/journal.pone.0225843 (PMC6894753; doi:10.1371/journal.pone.0225843)
Supplement: S1 File — (PDF) [file pone.0225843.s002.pdf]

HORSE.EGI	TIME	GROUP	ID
59.47247	0	C	CH2
93.2506	5	C	CH2
80.93593	10	C	CH2
75.00027	25	C	CH2
59.92181	45	C	CH2
91.74722	0	C	CH3
86.86092	5	C	CH3
93.85631	10	C	CH3
85.61136	25	C	CH3
132.7677	45	C	CH3
94.63655	0	C	CH4
97.91568	5	C	CH4
83.52916	10	C	CH4
95.16256	25	C	CH4
79.95145	45	C	CH4
128.3038	0	C	CH5
114.6298	5	C	CH5
130.285	10	C	CH5
106.5953	25	C	CH5
118.262	45	C	CH5
77.33557	0	T	TH1
76.09114	5	T	TH1
73.01028	10	T	TH1
88.77207	25	T	TH1
79.51493	45	T	TH1
95.69388	0	T	TH2
70.64051	5	T	TH2
84.55924	10	T	TH2
72.68906	25	T	TH2
80.49555	45	T	TH2
80.76802	0	T	TH3
105.4138	5	T	TH3
87.19148	10	T	TH3
102.8453	25	T	TH3
89.98752	45	T	TH3

Pony	Basal.egf	2h.egf	basal.ins	2h.ins	Status
1	114.0536	123.5384	10.6	432	ID
2	980.2285	525.2153	10.1	280	ID
3	57.68813	118.7761	6.88	540	ID
4	148.4774	112.8367	2	63.5	ID
5	52.3239	59.72247	2	65.7	H
7	76.92953	75.78766	2	20.2	H
8	129.9653	299.3938	2	208	ID
9	67.53555	66.86697	2	62.8	H
10	47.08068	45.47844	5.54	492	ID
11	122.8789	90.63255	2	32.6	H
12	206.4205	219.8557	2	37	H

13	90.69738	91.69143	2	11.7	H
14	99.39166	121.4613	2	211	ID
15	96.2236	77.31229	2	34.7	H
16	437.9642	415.6947	2	270	ID
18	881.7065	822.1113	5.53	444	ID

CH Phos	TH Phos
9198.297	11730.59
10684.14	13249.7
10937.03	11184.32
11979.91	

ID	EGFR copie	Tissue
CH1	16.06366	L
CH2	29.60948	L
CH3	14.95169	L
CH4	7.876224	L
CH5	57.38654	L
TH1	59.14757	L
TH2	20.32094	L
TH3	25.27776	L
TH4	5.660398	L
CH1	13.14577	S
CH2	58.13046	S
CH3	4.099329	S
CH4	35.61958	S
CH5	8.958475	S
TH1	10.21549	S
TH2	14.42756	S
TH3	13.4791	S
TH4	86.16073	S
